# Increased number and altered phenotype of lymphatic vessels in peripheral lung compartments of patients with COPD

**DOI:** 10.1186/1465-9921-14-65

**Published:** 2013-06-11

**Authors:** Michiko Mori, Cecilia K Andersson, Gerard J Graham, Claes-Göran Löfdahl, Jonas S Erjefält

**Affiliations:** 1 Unit of Airway Inflammation and Immunology, Department of Experimental Medical Sciences, Lund University, Lund, Sweden; 2 Department of Respiratory Medicine and Allergology, Skåne University Hospital, Lund University, Lund, Sweden; 3 Institute of Infection, Immunity and Inflammation, Glasgow Biomedical Research Centre, University of Glasgow, Glasgow, UK

**Keywords:** Chronic obstructive pulmonary disease, Alveolar, Lymphatic vessel, CCL21, D6, Immunohistochemistry, Inflammation

## Abstract

**Background:**

*De novo* lymphatic vessel formation has recently been observed in lungs of patients with moderate chronic obstructive pulmonary disease (COPD). However, the distribution of lymphatic vessel changes among the anatomical compartments of diseased lungs is unknown. Furthermore, information regarding the nature of lymphatic vessel alterations across different stages of COPD is missing. This study performs a detailed morphometric characterization of lymphatic vessels in major peripheral lung compartments of patients with different severities of COPD and investigates the lymphatic expression of molecules involved in immune cell trafficking.

**Methods:**

Peripheral lung resection samples obtained from patients with mild (GOLD stage I), moderate-severe (GOLD stage II-III), and very severe (GOLD stage IV) COPD were investigated for podoplanin-immunopositive lymphatic vessels in distinct peripheral lung compartments: bronchioles, pulmonary blood vessels and alveolar walls. Control subjects with normal lung function were divided into never smokers and smokers. Lymphatics were analysed by multiple morphological parameters, as well as for their expression of CCL21 and the chemokine scavenger receptor D6.

**Results:**

The number of lymphatics increased by 133% in the alveolar parenchyma in patients with advanced COPD compared with never-smoking controls (p < 0.05). In patchy fibrotic lesions the number of alveolar lymphatics increased 20-fold from non-fibrotic parenchyma in the same COPD patients. The absolute number of lymphatics per bronchiole and artery was increased in advanced COPD, but numbers were not different after normalization to tissue area. Increased numbers of CCL21- and D6-positive lymphatics were observed in the alveolar parenchyma in advanced COPD compared with controls (p < 0.01). Lymphatic vessels also displayed increased mean levels of immunoreactivity for CCL21 in the wall of bronchioles (p < 0.01) and bronchiole-associated arteries (p < 0.05), as well as the alveolar parenchyma (p < 0.001) in patients with advanced COPD compared with never-smoking controls. A similar increase in lymphatic D6 immunoreactivity was observed in bronchioles (p < 0.05) and alveolar parenchyma (p < 0.01).

**Conclusions:**

This study shows that severe stages of COPD is associated with increased numbers of alveolar lymphatic vessels and a change in lymphatic vessel phenotype in major peripheral lung compartments. This novel histopathological feature is suggested to have important implications for distal lung immune cell traffic in advanced COPD.

## Background

Chronic obstructive pulmonary disease (COPD) is a chronic, inflammatory lung disease with increased mortality and morbidity [1]. Long-term smoke exposure is the primary cause for developing COPD. The inflammatory reactions of airways, pulmonary vasculature, and alveolar parenchyma are related to both innate and adaptive immune responses [2-5]. For example, several studies have revealed increased numbers of lymphocytes [6,7], mast cells [8], dendritic cells [9,10], and ectopic lymphoid aggregates in peripheral lungs of patients with COPD [11-13].

The lymphatic vessels are, apart from their role in interstitial fluid homeostasis, critical for transporting antigen-loaded dendritic cells and memory/effector T-cells to draining lymph nodes where they initiate adaptive immune responses [14-16]. Recent studies suggest that naïve T-cells are also transported to lymph nodes via lymphatic vessels [17]. It is also likely that lung lymphatic vessels transport leukocytes to the ectopic lymphoid aggregates in COPD lungs.

The entry of antigen-loaded dendritic cells into lymphatic vessels and their migration to draining lymph nodes is regulated by the homeostatic CC chemokine ligand CCL21, also known as secondary lymphoid chemokine, which is secreted by the lymphatic endothelial cells and binds to its receptor (the CC chemokine receptor CCR7) on activated dendritic cells [18-21]. The lymphatic endothelium also expresses the chemokine scavenger receptor D6, which binds and degrades inflammatory CC chemokines [22-24]. Thus, D6 clears the lymphatic endothelial surface of inflammatory chemokines to maintain the chemoattractant gradient of CCL21 and facilitate the migration of CCR7-positive immune cells to lymph nodes [25].

Lymphatic vessels have been implicated in several pathological conditions of the lung. Formation of new lymphatic vessels (i.e. lymphangiogenesis) has been described in patients with lymphangioleiomyomatosis [26] and interstitial lung diseases, including idiopathic pulmonary fibrosis (IPF) [27] and diffuse alveolar damage [28]. In contrast, reduced number of lymphatic vessels has been reported in airways of patients who died of severe asthma [29]. Recent observations that lymphatics are closely associated with pulmonary sarcoid granulomas [30,31] further support a general involvement of lymphatic vessels in pulmonary diseases. In a mouse model of chronic airway inflammation, *Mycoplasma pulmonis* infection resulted in airway lymphangiogenesis which was mediated by VEGF-C and VEGF-D producing leukocytes [32]. In the same model, inhibition of lymphangiogenesis resulted in mucosal oedema. Only one previous study has been performed in patients with COPD [33]. This study by Hardavella et al. [33] provides an important indication that lymphatic vessels are more numerous in patients with moderate COPD. However, it remains unknown to what extent the lymphatic system is increased in patients with other severities of COPD. Other remaining questions concern the distribution of lymphatic vessel changes among the different compartments of the peripheral lung and whether the lymphatic endothelium has been phenotypically altered.

The aim of this study is to perform a detailed morphometric characterization of lung lymphatic vessels in patients with different severities of COPD and to assess disease-related changes across distinct peripheral lung compartments where the lymphatics have remained poorly studied; i.e. bronchioles, pulmonary arteries and alveolar parenchyma. Additional focus is on the lymphatic expression of key molecules involved in immune cell trafficking.

## Methods

### Patients and lung tissue collection

Lung resection specimens were obtained from never smokers, smokers and patients with GOLD stage I-III COPD undergoing therapeutic lung resection surgery for bronchial tumour. Only patients with well-delineated solitary tumours were included in the study. Care was taken to collect peripheral lung tissue as far from the tumour as possible to avoid cancer tissue in any of the sections, a procedure that repeatedly has been used to collect lung tissue samples [8,10,34]. Explant lungs from GOLD stage IV COPD patients, who lacked any history of lung cancer, were collected during lung transplantation surgery at Skåne University Hospital, Lund, Sweden. At the time of surgery, all patients were free from exacerbation. The tissue samples used in this study have, in part, been described in our previous publications [8,12]. Severity of COPD was classified according to GOLD criteria [1]. Patients with COPD (n = 27) were divided into three subgroups: patients with mild (GOLD stage I), moderate-severe (GOLD stage II-III) and very severe (GOLD stage IV) COPD. Non-COPD control subjects (n = 14) were divided into never smokers and ex-/current smokers without COPD. For more detailed subject characteristics, see Table 1. For each patient, peripheral tissue samples from randomly selected lobes were immersed into 4% buffered paraformaldehyde immediately after surgical excision and multiple paraffin-embedded tissue blocks were prepared for histological analysis. This study was approved by the Swedish Research Ethics Committee in Lund. All patients signed informed consent to participate in the study.

**Table 1:** Characteristics of the study subjects

**Parameters**	**Never smokers**	**Smokers w/o COPD**	**GOLD I COPD**	GOLD II-III * COPD	**GOLD IV COPD**	**Overall p-value**
Subjects, n	8	6	6	11	10	
Gender, male/female	2/6	3/3	4/2	9/2	4/6	
Age, years	66 (33–76) ^#^	55 (47–68)	68 (56–75)	73 (61–77) ^†^	62 (53–66) ^§^	<0.01
Height, m	1.6 (1.5-1.8)	1.8 (1.6-1.8)	1.8 (1.6-1.8)	1.7 (1.6-1.9)	1.7 (1.5-1.9)	0.247
Body mass index, kg/m^2^	22.6 (19.8-29.7)	22.2 (19.7-26.6)	23.1 (20.3-26.8)	25.3 (17.7-33.3)	22.9 (18.0-27.2)	0.462
Smoking history, pack-years	0	40 (20–80)	39 (25–66)	47 (16–65)	43 (25–60)	0.783
Smoking status, ex-smokers/current	NA	3/3	3/3	8/3	10/0	
FEV_1_, L	2.5 (1.7-5.1)	3.1 (1.9-3.5)	2.9 (1.6-3.2)	1.8 (1.2-2.3)	0.6 (0.4-1.0)	<0.001
FEV_1_/(F)VC, %	82 (66–121)	78 (71–88)	67 (65–70)	53 (41–67) ^≠^	32 (20–39)	<0.001
FEV_1_, % of predicted	109 (82–141)	93 (82–120)	86 (80–95)	64 (43–74) ^≠^	23 (15–27)	<0.001
Inhaled β_2_-agonists						
Short-acting (yes/no/unknown)	0/8/0	0/6/0	1/5/0	2/9/0	4/5/1^‡^	
Long-acting (yes/no/unknown)	0/8/0	0/6/0	0/6/0	0/11/0	4/5/1^‡^	
Inhaled anticholinergics						
Short-acting (yes/no/unknown)	0/8/0	0/6/0	1/5/0	2/9/0	3/6/1^‡^	
Long-acting (yes/no/unknown)	0/8/0	0/6/0	0/6/0	0/11/0	5/4/1^‡^	
Inhaled short-acting β_2_-agonist plus anticholinergics (yes/no/unknown)	0/8/0	0/6/0	0/6/0	0/11/0	3/6/1^‡^	
Corticosteroids						
Inhaled (yes/no/unknown)	0/8/0	0/6/0	0/6/0	1/10/0	2/7/1^‡^	
Oral (yes/no/unknown)	0/8/0	0/6/0	0/6/0	0/11/0	2/7/1^‡^	
Inhaled long-acting β_2_-agonist plus corticosteroids (yes/no/unknown)	0/8/0	0/6/0	0/6/0	1/10/0	7/2/1^‡^	
Mucolytics (yes/no/unknown)	0/8/0	0/6/0	2/4/0	0/11/0	5/4/1^‡^	

Values are median (range) or n. Statistical analysis was performed using Kruskal-Wallis nonparametric test followed by Dunn's multiple comparison post-test. COPD: chronic obstructive pulmonary disease; FEV_1_: forced expiratory volume in one second; (F)VC: (forced) vital capacity; GOLD: Global Initiative for Chronic Obstructive Lung Disease; NA: not applicable.

*: Two patients with GOLD stage III COPD (median value of FEV_1 _% of predicted, 44.5%; range 43.2-45.9).

^#^: The mean value of the study group is 63 years.

^‡^: One patient with unknown medical history.

^†^: p < 0.01 vs. smokers without COPD.

^§^: p < 0.05 vs. patients with GOLD stage II-III COPD.

: p < 0.001 vs. never smokers.

: p < 0.001 vs. smokers without COPD.

: p < 0.01 vs. patients with GOLD stage I COPD.

^≠^: p < 0.01 vs. never smokers.

: p < 0.05 vs. patients with GOLD stage I COPD.

### Immunohistochemistry

#### ***Validation of markers for proper identification of human lung lymphatic vessels***

Since there are still some remaining questions regarding the suitability of lymphatic markers to specifically detect lung lymphatic vessels, an initial aim was to validate the specificity of common antibodies against lymphatic endothelial cells. For this purpose we initiated our study with a careful evaluation of the previously published lymphatic vessel markers LYVE-1, podoplanin (D2-40), and Prox1 for their capacity to distinguish lung lymphatic vessels from pulmonary blood vessels (some of which are known to express lymphatic vessel markers [35]).

#### ***Protocols for immunohistochemistry***

Four-micron-thick paraffin-embedded sections were heated at 60°C for 20 min, and then subjected to simultaneous dewaxing and antigen retrieval according to Table 2. All staining procedures were performed in an automated slide stainer (Autostainer Plus, DakoCytomation, Glostrup, Denmark). Primary antibodies used are listed in Table 2.

**Table 2:** Primary antibodies used for immunohistochemistry

**Antigen**	Clone *	**Supplier**	**Antigen retrieval**	**Dilution**	**Against**
CD8	C8/144B	Dako, Glostrup, Denmark	Low pH ^#^	1:640	Cytotoxic T-cells
			PC		
CD11c	5D11	Novocastra/Leica, Newcastle Upon Tyne, UK	Low pH ^#^	1:100	Myeloid dendritic cells and macrophages
			PC		
CD20	L26	Dako	Low pH ^#^	1:1200	B-cells
			PC		
CD57	NK1	Novocastra/Leica	Low pH ^#^	1:200	Natural killer cells and T-cell subsets
			PC		
CD68	PG-M1	Dako	Low pH ^#^	1:600	Monocytes and macrophages
			PC		
Chemokine ligand 21 (CCL21)	Goat polyclonal	R&D Systems, Abingdon, UK	High pH^‡^	1:3000	Lymphatic endothelium
			PT		
Chemokine scavenger receptor D6	Made in-house [23]	Provided by Dr. G.J. Graham	High pH^‡^	1:400	Lymphatic endothelium, leukocytes
			PT		
Ki-67	Rabbit polyclonal	Biocare Medical, Concord, CA, USA	Low pH ^#^	1:200	Cell proliferation
			PC		
Lymphatic vessel endothelial hyaluronan receptor-1 (LYVE-1)	Rabbit polyclonal	Abcam, Cambridge, UK	Low pH ^#^	1:400	Lymphatic endothelium
			PC		
Podoplanin	D2-40	Biocare Medical	Low pH ^#^	1:100	Lymphatic endothelium
			PC		
Prospero-related homeobox transcription factor (Prox1)	Rabbit polyclonal	Abcam	High pH^‡^	1:800	Lymphatic endothelial cell nuclei
			PT		
α-smooth muscle actin (α-SMA)	1A4	Sigma-Aldrich, Saint Louis, MO, USA	Low pH ^#^	1:1000	Smooth muscle cells
			PC		

PC: pressure cook, 2100 Retriever, Prestige Medical Ltd., Blackburn, England; PT: pre-treatment module, Dako, Glostrup, Denmark.

*: Mouse monoclonal antibodies unless otherwise stated.

^#^: DIVA Decloaker, pH 6, DV2004 MX, Biocare Medical.

^‡^: EnVision™ FLEX Target Retrieval Solution, pH 9, K8004, Dako.

Single staining immunohistochemistry for LYVE-1 was performed using EnVision Peroxidase/Dab Detection System kit (Rabbit/mouse K5007, Dako, Glostrup, Denmark). Endogenous peroxidase activity was blocked with 0.3% hydrogen peroxide. After incubation with rabbit anti-human LYVE-1 polyclonal antibodies for 1h, sections were incubated with Polymer/HRP-linked secondary antibodies. Next, sections were incubated with 3,3’-diaminobenzidine (Dab) substrate solution for 10 min, counterstained with Mayer’s haematoxylin, dehydrated through ethanol series, cleared in xylene and mounted with Pertex (HistoLab, Gothenburg, Sweden).

Double staining immunohistochemistry was performed using EnVision G|2 Doublestain System (K5361, Dako). After blocking endogenous peroxidase and alkaline phosphatase activity, sections were incubated with antibodies directed against Prox1 or podoplanin for 1 h. Next, sections were incubated with Polymer/HRP-linked secondary antibodies followed by Dab substrate solution for 10 min. An additional blocking step with Dako Double Stain Blocking Reagent was performed to prevent additional binding of secondary antibodies to the first primary antibodies. Sections were then incubated with antibodies against podoplanin, CD8, CD11c, CD20, CD57, or CD68 for 1 h. Finally, sections were incubated with Polymer/AP-linked secondary antibodies, Dako Liquid Permanent Red substrate solution for 10 min, counterstained with Mayer’s haematoxylin, and mounted with Pertex.

Double staining immunohistochemistry for Prox1 and CCL21 or Prox1 and D6 was performed using an Avidin-Biotin/Streptavidin detection system. Sections were stained with rabbit anti-human Prox1 polyclonal antibodies using the protocol described for LYVE-1. After incubation with Dab substrate solution, sections were blocked with a denaturating solution (DNS001H, L, Biocare Medical, Concord, CA, USA) to prevent additional binding to the first primary antibody. Endogenous alkaline phosphatase activity was blocked with Dako Dual Enzyme Block (S2003). Sections were then incubated with Dako Protein Block Serum Free (X0909) and an additional blocking with Dako Avidin/Biotin Blocking solution (X0590) was performed. After incubation with antibodies against goat anti-human CCL21 or mouse anti-human D6, sections were incubated with biotinylated rabbit anti-goat (1:200, BA-1000, Vector Laboratories, Inc., Burlingame, CA, USA) or biotinylated horse anti-mouse IgG secondary antibodies (1:200, BA-2000, Vector Laboratories). Next, sections were incubated with Streptavidin-AP (1:100, D0396, Dako) and immunoreactivity was detected with Dako Liquid Permanent Red substrate solution (K0640). Finally, sections were counterstained with Mayer’s haematoxylin and mounted with Pertex.

Double staining immunohistochemistry for podoplanin and α-smooth muscle actin (α-SMA) was performed as described previously [12].

Immunostaining for podoplanin as well as CCL21 and D6 was performed in batches containing sections from control subjects and patients with COPD to avoid variations in staining intensity.

### Quantitative assessments of lymphatic vessels

To compare the anatomical distribution of lymphatic vessels among the study groups, tissue blocks containing bronchioles (non-cartilaginous, < 2 mm in cross-sectional internal diameter), pulmonary blood vessels, as well as alveolar parenchyma were included in the study. Thus, severely emphysematous tissue blocks, lacking bronchioles or pulmonary arteries, were excluded. A total of 62 tissue blocks (two blocks per subject) from control subjects and patients with GOLD stage I-III COPD and 30 large blocks (three blocks per subject) from patients with GOLD stage IV COPD was examined. In total 10 743 individual cross-sectioned lymphatic vessels were analysed across the study groups (total number of analysed lymphatic vessels in all tissue blocks were as follows: never smokers, 1457; smokers, 1034; GOLD stage I, 891; GOLD stage II-III, 1739; GOLD stage IV, 5622). For each section high-resolution digital images of whole lung tissue area were generated using a slide-scanning robot (ScanScope, Aperio Technologies, Vista, CA, USA). Morphometric and immunohistochemical measurements were performed on the generated digital images using Aperio ImageScope V.10.0 Image Analysis Software (Aperio Technologies). All quantifications were carried out on blinded sections.

On each section, all visible bronchioles and bronchiole-associated arteries, as well as multiple solitary blood vessels (at a distance from visible bronchioles and bronchiole-associated arteries) and large areas of alveolar tissue (at a distance from visible bronchioles, arteries, arterioles and veins) were investigated for the presence of podoplanin-immunopositive lymphatic vessels. The pleura or intra-lobular septa were not visible in all sections and, therefore, were excluded from the analysis. The area of the subepithelial tissue was measured by cursor tracing the region extending from the basement membrane to the parenchyma (i.e. the entire wall under the epithelium). The area of the adventitial layer of bronchiole-associated arteries was measured by manually tracing the region extending from the outer border of the tunica media to the alveolar parenchyma. Regions of non-emphysematous and non-fibrotic alveolar tissue were delineated (excluding bronchioles and blood vessels) and the pixels corresponding to the alveolar tissue (excluding airspaces) were counted and expressed as square mm of alveolar parenchyma. Solitary blood vessels were subdivided according to their mean cross-sectional internal lumen diameter, which was calculated by dividing the sum of the minimal and maximal endothelium-to-endothelium distance by two. The following subdivision was performed: <50 μm in mean diameter, 50–100 μm in mean diameter and >100 μm in mean diameter. The perimeter of each lymphatic vessel was delineated by cursor tracing along the lymphatic endothelium by freehand. To allow for a more complete picture of the nature of changes of the lymphatic vessels, these were analysed by four complementary parameters as described below.

#### ***Accumulated lymph vessel endothelial length per tissue area***

The accumulated lymphatic vessel endothelial length, calculated as the total sum of all lymphatic vessel perimeters, was normalized to the area of the bronchiolar subepithelial tissue, arterial adventitia or alveolar tissue.

#### ***Accumulated lymph vessel endothelial length per bronchiole or pulmonary vessel***

The total sum of all lymph vessel perimeters was calculated and data were expressed as the accumulated lymph vessel endothelial length per bronchiole, bronchiole-associated artery and solitary blood vessel. In addition, in bronchioles, the accumulated lymph vessel endothelial length was normalized to the length of the basement membrane perimeter.

#### ***Numbers of lymphatic vessels per tissue area***

The numbers of lymphatic vessels in each analysed tissue region were counted and data were normalized to the area of the bronchiolar subepithelial tissue, arterial adventitia, and alveolar tissue.

#### ***Numbers of lymphatic vessels per bronchiole or pulmonary vessel***

The numbers of lymphatic vessels were expressed per bronchiole, bronchiole-associated artery, and solitary blood vessel. In bronchioles, the numbers of lymph vessels were also normalized to the length of the basement membrane perimeter.

### Quantitative assessments of lymphatic CCL21 and D6 expression

Immunoreactivity for lymphatic CCL21 and D6 was quantified in the endothelium of Prox1-immunopositive lymphatic vessels in tissue sections from never-smoking controls and patients with GOLD stage IV COPD. The outer perimeter of the lymphatic endothelium was delineated and a fixed threshold for identification of red positive staining was set in order to only calculate the number of pixels corresponding to CCL21 or D6 immunoreactivity (Aperio Positive Pixel Count Algorithm V.9, Aperio Technologies). Pixels corresponding to Prox1 immunoreactivity (brown colour) were automatically excluded in the analysis. Data were expressed as lymphatic CCL21 or D6 immunoreactivity per unit area of tissue or per lymphatic vessel perimeter.

### Statistics

Statistical analysis was performed using GraphPad Prism V.5.0 (GraphPad software, San Diego, CA, USA). Kruskal-Wallis nonparametric test followed by Dunn’s multiple comparisons post-test was used for comparison between all study groups and Mann–Whitney rank sum test for comparison between two groups. A p value <0.05 was considered significant.

## Results

### Podoplanin is a suitable marker for lung lymphatic vessels

**Figure 1:**
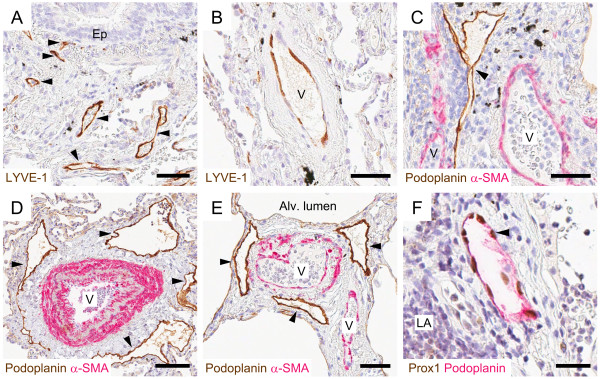
**Staining characteristics of immunohistochemical markers for detection of lymphatic vessels in the peripheral lung. **LYVE-1 immunoreactivity was present on vessels with **(A) **lymphatic morphology (brown, arrowheads) and **(B) **blood vessel morphology (brown). **(C**-**E) **Double staining immunohistochemistry for podoplanin (brown, arrowheads) and α-smooth muscle actin (α-SMA, red) revealed podoplanin-immunoreactivity on lymphatic vessels and not on α-SMA-positive blood vessels (V). **(F) **Double staining immunohistochemistry for podoplanin (red) and Prox1 (brown nuclei, exemplified by arrowhead). Ep, bronchiolar epithelium; LA, lymphoid aggregate. All sections were counterstained with Mayer’s haematoxylin (blue stain). Black endogenous pigment deposition is visible in **(A**-**C)**. *Scale bars: ***(A**-**C**, **E) **50 μm; **(D)** 100 μm; **(F) **25 μm.

**Figure 2:**
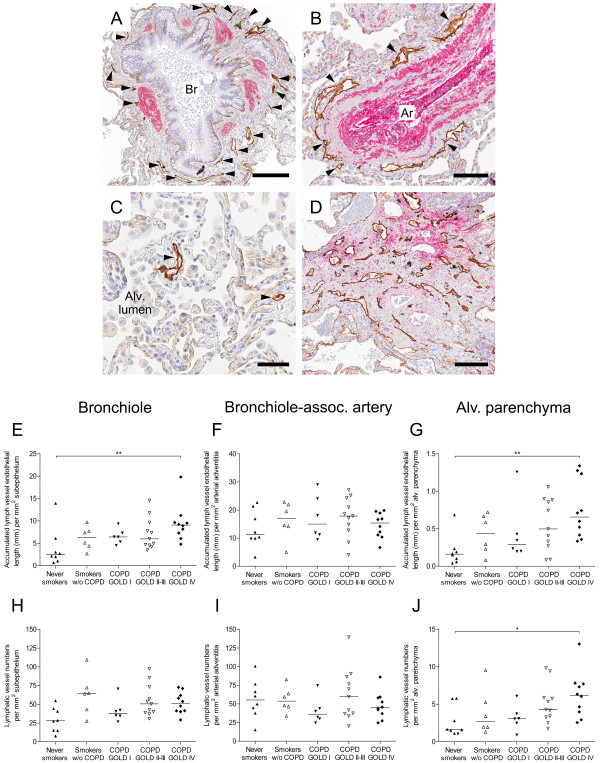
**Increased number of lymphatic vessels in the alveolar compartment in patients with advanced COPD. (A**-**D) **Immunohistochemical staining for podoplanin (brown, arrowheads) and α-smooth muscle actin (α-SMA, red) in sections of a patient with GOLD stage IV COPD. Lymphatic vessels are present in **(A)** bronchiolar (Br) subepithelial tissue, **(B) **adventitia of bronchiole-associated arteries (Ar), **(C) **alveolar parenchyma and **(D) **patchy fibrotic lesions. Cell nuclei were counterstained with Mayer’s haematoxylin (blue stain). **(E**-**G) **Accumulated lymphatic vessel endothelial length (i.e. the total sum of all lymphatic perimeters) in **(E) **bronchiolar subepithelial tissue, **(F)** adventitia of bronchiole-associated arteries and (**G**) non-fibrotic alveolar parenchyma in never smokers, smokers without COPD and patients with COPD. **(H**-**J) **Number of lymphatic vessels in **(H) **bronchiolar subepithelial tissue, **(I) **adventitia of bronchiole-associated arteries and **(J) **non-fibrotic alveolar parenchyma in never smokers, smokers without COPD and patients with COPD. Statistical analysis was performed using Kruskal-Wallis nonparametric test and Dunn’s multiple comparison post-test. Horizontal lines indicate median value. * p < 0.05; ** p < 0.01. *Scale bars: ***(A)** 200 μm; **(B) **100 μm; **(C) **50 μm; **(D) **150 μm.

In initial studies antibodies against LYVE-1 and podoplanin were evaluated for proper identification of lymphatic vessels in routine paraffin-embedded human lung sections. Immunoreactivity for both markers was detected on vessels with an irregular endothelial morphology, thin vessel wall, and sometimes barely visible lumen (Figure 1A and 1C). Whereas LYVE-1 immunoreactivity was occasionally detected also on scattered blood vessels (Figure 1B), podoplanin was restricted to vessels that lacked immunoreactivity for blood vessel-associated α-SMA (Figure 1C-E). Co-expression analysis of podoplanin and Prox1, a transcription factor expressed in lymphatic endothelial cell nuclei, further confirmed podoplanin-positive vessels to be true lymphatic vessels (Figure 1F). In addition to lymphatic vessels, weak immunoreactivity for podoplanin was detectable on Type I pneumocytes (exemplified in Figure 1D-E and Figure 2A-C) and epithelial basal cells (exemplified in Figure 2A). However, this non-lymphatic staining could easily be excluded in our quantification and assessment of lymphatics. Hence, podoplanin was used for further detection of lung lymphatic vessels.

### Localization of lymphatic vessels in peripheral lung compartments

In all study groups, lymphatic vessels were present in the wall of bronchioles and bronchiole-associated arteries, as well as in the region between a bronchiole and an artery (i.e. bronchiolar-vascular bundle). In the bronchiolar wall, lymphatic vessels were foremost present in the adventitia layer and to a lesser extent in the lamina propria (exemplified in Figure 2A). For all study groups, lymphatic vessels in bronchiolar-vascular bundles generally had a greater perimeter compared to lymphatics associated with the subepithelial tissue. In agreement with previous studies in the lung [36], the lymphatic vessels in bronchiole-associated arteries were restricted to the adventitia layer (Figure 2B). Also, α-SMA-positive solitary blood vessels (well-separated from bronchioles) were associated with adventitial lymphatics (exemplified in Figure 1D-E). Intra-alveolar lymphatic vessels, not associated with α-SMA-positive blood vessels or any conducting airways, were also found in all study groups (exemplified in Figure 2C).

### Differential lymphatic changes among peripheral lung compartments in patients with advanced COPD

#### ***Bronchioles***

In patients with GOLD stage IV COPD the accumulated lymph vessel endothelial length in the subepithelial tissue was significantly increased compared with never-smoking and smoking control subjects (Figure 2E and Table 3). Whereas the absolute numbers of lymphatic vessels (expressed as lymphatic vessel numbers per bronchiole or basement membrane perimeter) were increased in patients with GOLD stage IV COPD compared with never-smoking controls (Table 3), no statistical differences were observed when numbers were normalized to the unit area of the subepithelial tissue (Figure 2H).

**Table 3:** Quantitative data on lymphatic vessel parameters

**Parameters**	**Never smokers**	**Smokers w/o COPD**	**GOLD I COPD**	**GOLD II-III COPD**	**GOLD IV COPD**	**Overall p-value**
**Bronchiole**						
Lymphatic vessel numbers per mm BM	1.91	4.09 *	2.94	3.76 *	4.11 *	<0.001
	(0.40-2.92)	(3.11-6.42)	(2.13-6.78)	(3.18-6.68)	(2.18-5.23)	
Lymphatic vessel numbers per bronchiole	3.13	5.33	6.88	7.83	10.85 *	<0.01
	(0.50-8.47)	(3.00-10.78)	(2.00-14.50)	(4.00-13.00)	(6.42-21.33)	
Accumulated lymphatic vessel endothelial length (mm) per mm BM	0.19	0.44	0.54	0.50	0.75 *	<0.01
	(0.04-0.68)	(0.38-0.56)	(0.33-0.93)	(0.30-0.87)	(0.42-1.01)	
Accumulated lymphatic vessel endothelial length (mm) per bronchiole	0.58	0.67	1.05	0.80	1.94* ^#^	<0.001
	(0.04-1.09)	(0.31-0.94)	(0.42-1.96)	(0.41-1.91)	(0.78-3.94)	
**Bronchiole-associated artery**						
Lymphatic vessel numbers per artery	3.78	4.17	3.50	5.50	8.07 * ^§^	<0.01
	(0.50-8.50)	(4.00-7.67)	(2.80-9.00)	(1.67-10.00)	(4.10-10.11)	
Accumulated lymphatic vessel endothelial length (mm) per artery	0.97	1.53	1.38	1.59	2.39 *	<0.05
	(0.11-3.32)	(0.60-1.91)	(0.95-2.90)	(0.60-2.60)	(1.39-4.66)	
**Alveolar parenchyma**						
Lymphatic vessel numbers per mm^2^ fibrotic parenchymal lesions	NA	NA	NA	NA	117.8 ^‡^	-
					(101.1-134.4)	

Values are median (range). Statistical analysis was performed using Kruskal-Wallis nonparametric test followed by Dunn's multiple comparison post-test. COPD: chronic obstructive pulmonary disease; BM: basement membrane; GOLD: Global Initiative for Chronic Obstructive Lung Disease; NA: not applicable.

*: p < 0.01 vs. never smokers.

^#^: p < 0.01 vs. smokers without COPD.

^§:^ p < 0.05 vs. patients with GOLD stage I COPD.

^‡^: Two patients with GOLD stage IV COPD.

#### ***Bronchiole-associated arteries***

The accumulated lymph vessel endothelial length per artery was increased in patients with GOLD stage IV COPD compared with never-smoking controls (Table 3). However, no difference in the accumulated lymph vessel endothelial length per unit adventitial area was observed among the study groups (Figure 2F). Similarly, the lymphatic vessel numbers per artery was increased in patients with GOLD stage IV COPD compared with never-smoking controls and patients with GOLD stage I COPD (Table 3), but the numbers per unit adventitial area was not changed among the study groups (Figure 2I).

#### ***Alveolar parenchyma***

Intra-alveolar lymphatic vessels were scarce in never-smoking control subjects but increased both in terms of accumulated lymph vessel endothelial length and lymphatic vessel numbers in patients with GOLD stage IV COPD (Figure 2G and 2J). Moreover, two patients with GOLD stage IV COPD had identifiable fibrotic lesions in the alveolar parenchyma (exemplified in Figure 2D). A separate analysis of these fibrotic regions revealed that the numbers of lymphatic vessels were dramatically increased compared to the surrounding non-fibrotic alveolar tissue (Table 3).

#### ***Solitary pulmonary blood vessels***

**Figure 3:**
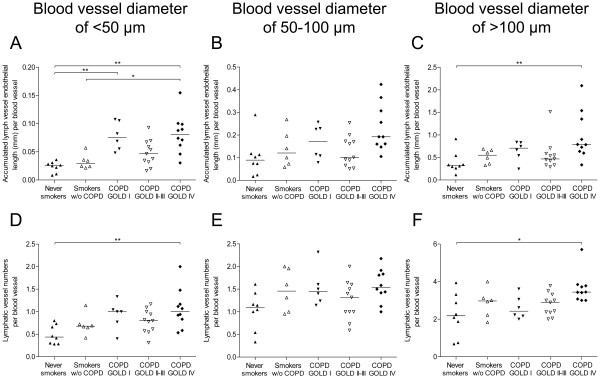
**Accumulated lymphatic endothelial length in solitary non-bronchiolar blood vessels. (A**-**C) **Accumulated lymphatic vessel endothelial length (i.e. the total sum of all lymphatic perimeters) and **(D**-**F) **number of lymphatic vessels associated with α-smooth muscle actin-positive solitary blood vessels with a diameter of < 50 μm, 50–100 μm and >100 μm in never smokers, smokers and patients with COPD. Statistical analysis was performed using Kruskal-Wallis nonparametric test and Dunn’s multiple comparison post-test. Horizontal lines indicate median value. * p < 0.05; ** p < 0.01.

In α-SMA-positive solitary blood vessels, both the accumulated lymph vessel endothelial length and lymphatic vessel numbers increased with increasing blood vessel diameter (Figure 3). In patients with GOLD stage IV COPD, both the accumulated lymph vessel endothelial length and numbers were increased in blood vessels of <50 μm in diameter compared with never-smoking and smoking control subjects (Figure 3A and 3D). A similar increase in the accumulated lymph vessel endothelial length was observed in patients with GOLD stage I compared with never-smoking controls (Figure 3A). In GOLD stage IV COPD, the accumulated lymph vessel endothelial length and numbers further increased in blood vessels of >100 μm in diameter compared with never-smoking control subjects (Figure 3C and 3F).

Importantly, in all lung compartments investigated only scarce number of lymphatic vessels showed immunoreactivity for the proliferation marker Ki-67 (data not shown).

### Size distribution of lymphatic vessels in peripheral lung compartments

**Figure 4:**
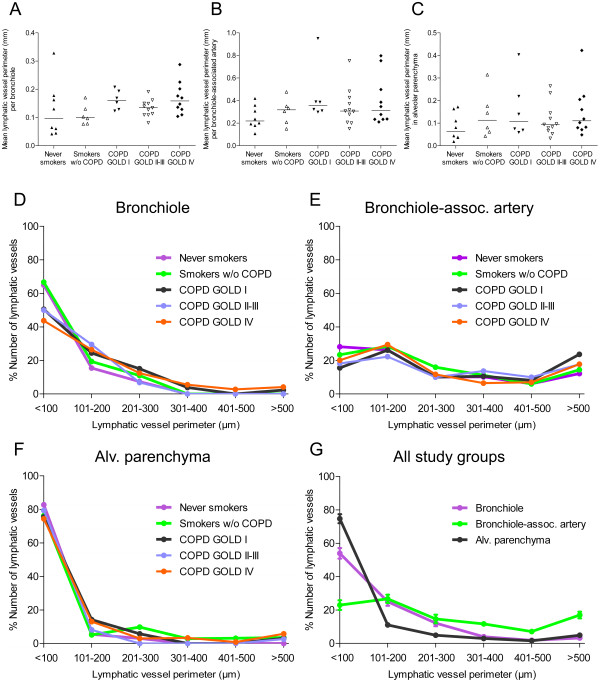
**Lymphatic vessels display similar size distribution among the study groups. (A**-**C) **Mean perimeter of individual lymphatic vessels in **(A) **bronchiolar subepithelial tissue, **(B) **adventitia of bronchiole-associated arteries and **(C)** alveolar parenchyma in never smokers, smokers and patients with COPD. Horizontal lines indicate median value. **(D**-**F) **Size distribution of individual lymphatic vessels in **(D) **bronchiolar subepithelial tissue (n = 2491), **(E) **adventitia of bronchiole-associated arteries (n = 1169) and **(F) **alveolar parenchyma (n = 2073) in control subjects and patients with COPD. Each point represents the median percentage of the number of lymphatic vessels within each specific vessel perimeter range. **(G) **Size distribution of all lymphatic vessels in bronchiolar subepithelial tissue, adventitia of bronchiole-associated arteries and alveolar parenchyma when study groups are combined. Each point indicates the mean, and error bars indicate SEM.

No differences in mean perimeter of individual lymphatic vessels were observed among the study groups (Figure 4A-C). Our assessment of thousands of individual lymphatic vessels per compartment allowed a detailed frequency analysis of the size distribution of lymphatic vessels. This analysis revealed that on the whole there was no clear difference in size distribution among the patient cohorts (Figure 4D-F). In bronchioles, however, there was a tendency for a shift towards larger lymphatic vessel sizes in the COPD groups (that in the 101 to 200 μm range also reached statistical significance for patients with GOLD stages II-IV COPD; overall p-value = 0.043) (Figure 4D). In bronchiole-associated arteries and alveolar parenchyma, no such differences in the lymphatic vessel size distribution were observed (Figure 4E-F). This analysis also showed that lymphatic vessels in the alveolar parenchyma were generally smaller than in bronchioles and associated arteries, the latter compartment having the largest mean perimeter (Figure 4G).

### Lymphatic vessels have an altered expression profile of CCL21 and D6 in advanced COPD

**Figure 5:**
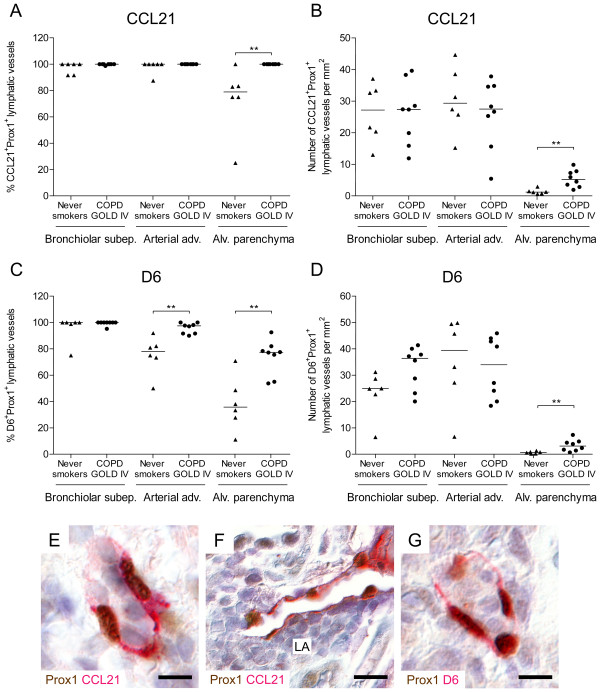
**Increased number of alveolar CCL21- and D6-positive lymphatic vessels in patients with advanced COPD. (A) **Percentage of CCL21-positive lymphatic vessels among all Prox1-positive lymphatics and **(B) **number of CCL21-positive lymphatic vessels in bronchiolar subepithelial tissue, adventitia of bronchiole-associated arteries and alveolar parenchyma in never smokers and patients with GOLD stage IV COPD. **(C)** Percentage of D6-positive lymphatic vessels among all Prox1-positive lymphatics and **(D) **number of D6-positive lymphatic vessels in similar lung compartments as for **(A **and **B) **in never smokers and patients with GOLD stage IV COPD. Mann–Whitney rank sum test was used for comparison between two groups. Horizontal lines indicate median value for each cohort. ** p < 0.01. **(E **and **F)** Immunohistochemical staining for CCL21 (red) and Prox1 (brown nuclei) and **(G)** immunohistochemical staining for D6 (red) and Prox1 (brown nuclei) in sections of patients with GOLD stage IV COPD. LA, lymphoid aggregate. Sections were counterstained with Mayer’s haematoxylin (blue stain). *Scale bars: ***(E**, **G)** 20 μm; **(F) **30 μm.

Since lymphatic vessels were increased in numbers in peripheral lungs of patients with advanced COPD we next investigated if these changes were also associated with altered lymphatic endothelial expression of molecules known to regulate leukocyte entry into the lymphatics. For this purpose we analysed the immunoreactivity for the homeostatic leukocyte chemoattractant chemokine CCL21 and the inflammatory chemokine scavenger receptor D6 on lymphatic vessels. The percentage of CCL21-immunopositive vessels among total Prox1-positive lymphatics was not changed in bronchioles and bronchiole-associated arteries, but significantly increased in the alveolar parenchyma in patients with GOLD stage IV COPD compared with never-smoking controls (Figure 5A). This was accompanied by increased absolute numbers of CCL21-positive lymphatic vessels in alveolar walls (Figure 5B). Patients with COPD had also increased percentages of D6-immunpositive vessels among all Prox1-positive lymphatics in both bronchiole-associated arteries and alveolar parenchyma (Figure 5C). However, the absolute number of D6-immunopositive lymphatic vessels was only increased in the alveolar parenchyma (Figure 5D). Immunostainings for lymphatic CCL21 and D6 are exemplified in Figure 5E-G. Lung lymphoid aggregates were also found to contain CCL21-immunopositive lymphatic vessels (Figure 5F).

**Figure 6:**
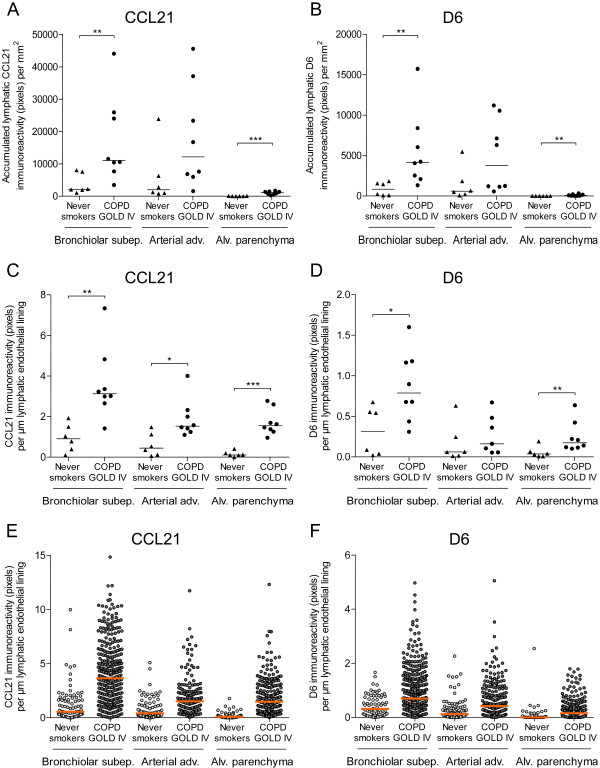
**Increased immunoreactivity for CCL21 and D6 in lymphatic endothelium in patients with advanced COPD. (A) **Accumulated lymphatic immunoreactivity of CCL21 and **(B) **D6 in bronchiolar subepithelial tissue, adventitia of bronchiole-associated arteries and alveolar parenchyma in never smokers and patients with GOLD stage IV COPD. Each point indicates the total amount of lymphatic immunoreactivity per unit area. **(C) **Immunoreactivity of CCL21 and **(D) **D6 per lymphatic vessel endothelial length in bronchiolar subepithelial tissue, adventitia of bronchiole-associated arteries and alveolar parenchyma. **(E **and **F) **Scatter graphs representing the immunoreactivity for **(E) **CCL21 and **(F) **D6 in each analysed lymphatic vessel in **(C **and **D)**. Mann–Whitney rank sum test was used for comparison between two groups. Horizontal lines indicate median value for each cohort. * p < 0.05; ** p < 0.01; *** p < 0.001.

Next, we investigated the accumulated amount of lymphatic staining for CCL21 and D6. Patients with GOLD stage IV COPD showed, compared with never-smoking controls, increased accumulated immunoreactivity for CCL21 as well as D6 (expressed as the total amount of lymphatic immunoreactivity per unit area) in bronchioles and alveolar parenchyma (Figure 6A-B). Analysis of all individual Prox1-immunopositive lymph vessels revealed that lymphatic CCL21 immunoreactivity (expressed as the total amount of immunoreactivity per lymphatic vessel endothelial length) was increased in all lung compartments; i.e. bronchioles, bronchiole-associated arteries, and alveolar parenchyma (Figure 6C and 6E). The immunoreactivity for lymphatic D6 was also increased on Prox1-immunopositive lymphatic vessels in patients with GOLD stage IV COPD (Figure 6D and 6F). This increase was restricted to bronchioles and alveolar parenchyma.

### Identification of leukocyte-containing lymphatic vessels

**Figure 7:**
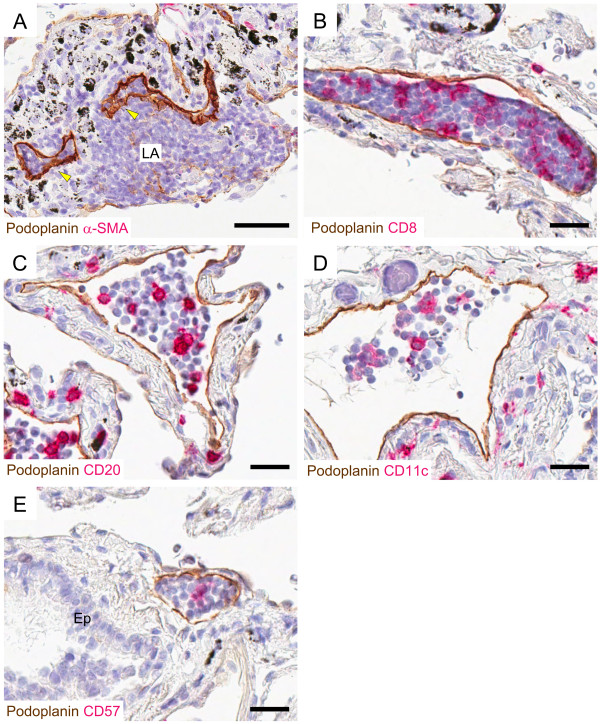
**Lymphatic vessels co-localize with lymphoid aggregates and contain immune cells. (A-E) **Micrographs from patients with GOLD stage IV COPD. **(A)** Lymphatic vessels (brown, arrowheads) frequently co-localized with lymphoid aggregates (LA). Double staining immunohistochemistry revealed that lymphatic vessels (brown) contain **(B) **CD8-positve T-cells, **(C) **CD20-positive B-cells, **(D)** CD11c-positive myeloid-like dendritic cells, and **(E) **CD57-positive NK/T-cells. In all sections, nuclei were counterstained with Mayer’s haematoxylin (blue stain). Black endogenous pigment deposition is visible in **(A-E)**. Ep, bronchiolar epithelium. *Scale bars: ***(A)** 50 μm; **(B-E) **25 μm.

As recently described [12], lymphatic vessels were frequently associated with lung lymphoid aggregates. In addition, across all patient cohorts, lymphatic vessels containing luminal mononuclear cells were observed in all lung compartments, including lymphoid aggregates (Figure 7). In patients with advanced COPD, several lymphatics were completely filled by cells (exemplified in Figure 7A-B and 7E). This finding was almost absent in control subjects. Double-staining immunohistochemistry tentatively identified the luminal cells (Figure 7B-E). Among the intra-lymphatic leukocytes were abundant CD8-positive T-cells and CD11c-positive myeloid-like dendritic cells as well as scattered CD20-positive B-cells and CD57-positive NK/T-cells. Luminal CD68-positive monocytes/macrophages were not detected in the lymphatic vessels.

## Discussion

This study reveals the presence and distribution of lymphatic vessels in peripheral compartments of human normal lungs and shows for the first time that in advanced COPD the most manifest increase in lymphatic numbers takes place in the alveolar compartment. This study further shows that in advanced COPD lymphatic vessels exhibit an altered phenotype characterized by up-regulation of CCL21 and D6. Considering the role of lymphatics in transporting leukocytes, these data suggest that lymphatic vessel changes may have important roles in the immunopathology of patients with severe stages of COPD.

The morphological evaluation of lymphatic vessel changes in the present study revealed further that the absolute numbers of bronchiolar and arterial wall lymphatic vessels are increased in lungs of patients with severe COPD compared with control subjects. However, normalization to tissue area did not reveal a similar increase in lymphatic vessel numbers. These results suggest that in these compartments tissue remodelling, such as thickening of the airway and arterial adventitia, is accompanied by a proportionally increased formation of lymphatic vessels. In bronchioles lymphatic vessel changes may also involve formation of more folded lymphatic vessels since the accumulated lymph endothelial length per area unit was increased in this compartment. The most clear lymphatic vessel changes were, however, observed in the alveolar parenchyma, a compartment where under normal conditions the lymphatics are relatively sparse in numbers.

The present type of morphological lymph vessel changes, which were most pronounced in advanced stage COPD, is likely to have several implications for the immunopathology and pathophysiology observed in this disease. As COPD becomes severe, inflammatory processes are significantly amplified [2]. Thus, the increased numbers of lymphatic vessels in advanced COPD could result from an increased demand for clearance of senescent leukocytes and extravasated fluid from the inflamed peripheral lung regions. The observed increased numbers of lymphatic vessels in this and a recent publication [33] may also reflect an increased demand for transporting activated immune cells from the sites of inflammation to draining lymph nodes. In support of this, this study reveals that the lymphatic endothelial expression of the chemokine CCL21 is increased in all peripheral lung compartments of patients with severe COPD. Among the major cells expressing the receptor for CCL21 are antigen-activated dendritic cells [20,37]. In vitro studies have shown that although CCR7 expression on dendritic cells may decrease in response to tobacco smoke, their capacity to migrate towards CCL21 is preserved [38]. Thus, the increased expression of CCL21, which is critical for the entry of immune cells into the lymphatic vessels, may facilitate the migration of activated dendritic cells [39] and enhance antigen presentation in draining lymph nodes. In addition to CCL21, our data also show that the lymphatic expression of D6 was increased in advanced COPD. Recent studies have suggested that lymphatic D6, which scavenges inflammatory CC chemokines, controls the flow of fluid and migration of appropriate immune cells to lymph nodes [25]. Previous studies have also demonstrated that D6 is expressed on leukocytes [40]. Interestingly, patients with COPD have increased percentage of D6-positive alveolar macrophages [41] indicating that D6 may generally be up-regulated in COPD lungs. Even though D6 is mainly expressed in lymphatic endothelium, this study confirms previous observations [42] that D6 is not expressed in all lymphatic vessels. It has been proposed that the lymphatic expression of D6 is up-regulated by pro-inflammatory cytokines [42]. It is likely that this mechanism is partly responsible for the presently observed up-regulation in COPD lungs. Interestingly, the mechanisms of up-regulation may also differ between the anatomical regions of the lung. For example, in the bronchioles almost 100% of the lymphatic vessels expressed D6 already in control lungs. Hence, in this compartment the increase in total lymph vessels-associated D6 in COPD may mainly be a consequence of a general increase in vessel numbers. On the other hand, in the alveolar parenchyma where only around 40% of the lymphatic vessels expressed D6 under baseline conditions, the up-regulation of D6 immunoreactivity in COPD lungs was also caused by a significant increase in the proportion of vessels expressing D6 (which in COPD was increased to around 80%). A similar increased proportion of positive alveolar lymphatic vessels was also observed for CCL21. Taken together, the combined action of increased lymphatic expression of D6 and CCL21 and a general increased density of lymphatic vessels in COPD lungs is likely to increase the capacity for regulated trafficking of leukocytes from the distal parts of the lung to draining lymph nodes.

This study further confirmed a close proximity between lymphatic vessels and ectopic lung lymphoid aggregates [12,43]. In addition to transporting leukocytes from the lung to draining lymph nodes, lymphatic vessels may efficiently transport leukocytes, as well as antigens, to local lymphoid aggregates in COPD lungs. Since we observed CCL21-immunoreactive lymphatic vessels within lymphoid aggregates, one could also speculate that these lymphatic vessels may offer an important route for leukocytes to exit from the lymphoid aggregates. Similar to lymph nodes, the lymphoid aggregates in COPD lungs contain lymphocytes, follicular dendritic cells and germinal centres [11,13,43] and are, thus, capable of initiating adaptive immune responses locally in the lung [44].

The molecular mechanism behind the increased number of lymphatics in COPD is currently unknown. It is of note, however, that immune cells such as macrophages may contribute to lymphatic vessel formation by their production of lymphangiogenic factors, including VEGF-C and VEGF-D [32,45,46]. Pro-inflammatory cytokines, many of which are up-regulated in COPD, such as TNF-α, can also regulate lymphatic vessel growth [47,48].

At a more upstream level respiratory infections may also induce the formation of lymphatics. Elegant studies in animal models of chronic airway inflammation have demonstrated that lymphangiogenesis, as well as remodelling of lymphatic vessels, occur extensively in lungs infected with *Mycoplasma pulmonis*[32,49]. Although other pathogens are generally associated with COPD, the majority of patients with advanced COPD suffer from recurrent infections of the lower respiratory tract [50] and this may be one of the driving forces for the presently observed peripheral lymphatic vessel changes in this patient category. In any case, an expanded lung lymphatic system is likely to result in a faster clearance of pathogens. Thus, the increased number of lymphatic vessels in major lung compartments in COPD may be a double-edged sword; a tool for accelerated adaptive immune responses in response to infections, and a structural basis for an aggravated inflammation in COPD lungs.

It is of note that once established an expanded lymphatic system may persist for considerable time [32]. Interestingly, after *Mycoplasma pulmonis* infection, remodelled blood vessels, in contrast to lymphatics, normalized readily once the underlying inflammation resolved [32]. This observation suggests that remodelling of the lymphatic system should be expected to be long lasting and relatively resistant to anti-inflammatory treatment. This notion is supported by the fact that lymphatic vessel alterations due to infections are steroid resistant [49]. In this study lymphatic vessel changes were most pronounced in patients who received inhaled corticosteroids indicating that also in human an expanded lymphatic system may not normalize readily upon steroid treatment. Future studies in larger COPD cohorts are, however, warranted to elucidate the detailed effects of steroids on newly formed lung lymphatics.

Although lymphatic vessel changes have been demonstrated in several lung diseases [33,51], it remains unknown if *de novo* formed lymphatics are fully functional in terms of interstitial fluid clearance. For example, in vivo animal studies have shown that newly formed lymphatics in inflammation have an altered structural phenotype that could impair clearance of fluid [52,53]. Whether or not the increased number of lymphatics in COPD lungs sufficiently compensates increased plasma leakage in, for example, an exacerbation remains to be investigated.

As indicated by this study, the role, extent and type of lymph vessel change may differ between the different compartments of the lung. Indeed, our data suggest that the relative increase in numbers and activation differed between bronchioles, vessels and the alveolar parenchyma. Unfortunately, our study material did not contain enough pleural material to justify a meaningful quantitative analysis in this compartment, which normally is rich in lymphatic vessels. Nevertheless, we did analyse other microenvironments such as patchy fibrotic lesions that can be observed in advanced COPD. Confirming other studies [27,28] our study revealed a marked increase of lymphatic vessels in fibrotic lesions. Interestingly, while newly formed lymphatic vessels normally develop from existing ones through sprouting, in fibrosis some of the newly formed lymphatic vessels lack connection to existing lymphatics and may thus have a different growth pattern [27,28]. Whether similar mechanism for growth of lymphatic vessels are active in the fibrotic lesions that develop in advanced stages of COPD remains to be investigated.

Although conflicting data exist regarding the presence of lymphatic vessels in alveolar parenchyma [27,28,54], alveolar podoplanin-immunoreactive lymphatic vessels have been detected in normal human lungs [36,55]. Also in this study we could observe lymphatic vessels in the alveolar parenchyma. However, it cannot be excluded that some of the alveolar lymphatic vessels may be associated with pulmonary blood vessels not visible by the 2D-projections provided by conventional histological sections. Also, in our 2D analysis it cannot be excluded that the increased numbers of lymphatic vessels may to some extent be caused by the folding of vessels into complex 3D-structures. The overall orientation of blood vessels and bronchioles was in the present study, however, random and equal among the study groups. Therefore, the presently observed differences in lymphatic vessel changes are unlikely a result from biased tissue orientation.

In this study patients with suspected lung cancer were included. This may be relevant information when investigating lung lymphatic vessels as lymphangiogenesis is of importance in tumour metastasis [56]. Lung tissue specimens used in this study were, however, obtained as far away from the tumour as possible in order to minimize the risk of cancer as potential contributor to the expanded lymphatic system revealed in COPD. Two factors strongly argue that this is not the case. Firstly, the patients of our non-COPD control groups had similar types of solid tumours, suggesting that all differences in the COPD-group are truly COPD related. Secondly, increased lymphatic vessel numbers were detected in patients with very severe COPD who lacked any history of cancer and where the tissue was collected in association with lung transplantation. As most histological human studies, another limitation of this study was the relatively small study groups, a factor that we in this study have tried to compensate by exploring several lung regions, each containing multiple lung compartments, in each study subject.

## Conclusions

In summary, this study demonstrates that in advanced stages of COPD lymphatic vessels are not only increased in numbers in the peripheral lung, they are also phenotypically altered with an increased expression of lymphatic vessel-associated CCL21 and D6. These novel findings are suggested to have implications for immune cell traffic in lungs of patients with advanced stages of COPD.

## Abbreviations

CCL21: CC chemokine ligand 21; CCR7: CC chemokine receptor 7; COPD: Chronic obstructive pulmonary disease; Dab: 3,3’-diaminobenzidine; FEV1: Forced expiratory volume in 1 second; FVC: Forced vital capacity; GOLD: Global Initiative for Chronic Obstructive Lung Disease; IPF: Idiopathic pulmonary fibrosis; α-SMA: α-smooth muscle actin.

## Competing interests

The authors declare that they have no competing interests.

## Authors’ contributions

MM collected tissue samples, performed laboratory work, quantified all immunostainings, performed statistical analysis, interpreted the data, and wrote the manuscript. CKA contributed to collection and handling of tissue samples, clinical characterization and revision of the manuscript. GJG helped with data interpretation and revised the manuscript. CGL contributed to the clinical patient characterization. JSE designed and supervised the study, interpreted the data and critically revised the manuscript. All authors approved the final version of the manuscript.
